# Influence of Mesalazine on Ferroptosis-Related Gene Expression in In Vitro Colorectal Cancer Culture

**DOI:** 10.3390/biomedicines13010219

**Published:** 2025-01-16

**Authors:** Joanna Słoka, Barbara Strzałka-Mrozik, Sebastian Kubica, Ilona Nowak, Celina Kruszniewska-Rajs

**Affiliations:** Department of Molecular Biology, Faculty of Pharmaceutical Sciences in Sosnowiec, Medical University of Silesia, 40-055 Katowice, Poland; joanna.sloka13@gmail.com (J.S.); sebastian.kubica@sum.edu.pl (S.K.); mc.ilona.nowak@gmail.com (I.N.); ckruszniewska@sum.edu.pl (C.K.-R.)

**Keywords:** mesalazine, ferroptosis, colorectal cancer, gene expression, oligonucleotide microarray

## Abstract

**Background/Objectives:** Colorectal cancer (CRC) is one of the most common oncological disorders. Its fundamental treatments include surgery and chemotherapy, predominantly utilizing 5-fluorouracil (5-FU). Despite medical advances, CRC continues to present a high risk of recurrence, metastasis and low survival rates. Consequently, significant emphasis has been directed towards exploring novel types of cell death, particularly ferroptosis. Ferroptosis is characterized by iron imbalance and the accumulation of lipid peroxides and reactive oxygen species (ROS), leading to cellular damage and death. Thus, the discovery of safe inducers of ferroptosis, offering new hope in the struggle against CRC, remains crucial. In this study, we applied the concept of drug repositioning, selecting mesalazine (MES), a non-steroidal anti-inflammatory drug (NSAID), for investigation. **Methods:** The study was conducted on the colon cancer cell line DLD-1 and normal intestinal epithelial cells from the CCD 841 CoN cell line. Both cell lines were treated with MES solutions at concentrations of 10, 20, 30, 40, and 50 mM. Cytotoxicity was assessed using the MTT assay, while ferroptosis-related gene expression analysis was performed using oligonucleotide microarrays, with RT-qPCR used for validation. **Results:** MES effectively reduces the viability of DLD-1 cells while minimally affecting normal intestinal cells. Subsequent oligonucleotide microarray analysis revealed that MES significantly alters the expression of 56 genes associated with ferroptosis. **Conclusions:** Our results suggest that MES may induce ferroptosis in CRC, providing a foundation for further research in this area.

## 1. Introduction

Programmed cell death (PCD) is fundamental to processes such as embryogenesis, tissue homeostasis and the immune response. It plays a crucial role in normal development and prevents hyperproliferative diseases such as cancer [1]. Researchers have identified various types of PCD including apoptosis, pyroptosis, parthanatos, necroptosis and ferroptosis [2].

Ferroptosis is a recently discovered form of regulated cell death, characterized by intracellular phospholipid peroxidation. This unique mechanism of cell death is morphologically, biologically and genetically distinct from other types. Unlike apoptosis, which is generally non-inflammatory and involves well-organized cellular dismantling, ferroptosis is associated with the accumulation of lipid peroxides and ROS, leading to cellular damage and death [3].

The central mechanism driving ferroptosis involves the peroxidation of polyunsaturated fatty acids (PUFAs) within membrane phospholipids. This lipid peroxidation is catalyzed by ROS, generated in the presence of free iron via the Fenton reaction. Elevated intracellular iron levels, often resulting from disrupted iron homeostasis, exacerbate oxidative stress and increase susceptibility to ferroptosis [4].

A key regulator of ferroptosis is glutathione peroxidase 4 (GPX4), an enzyme that detoxifies lipid peroxides. GPX4 activity depends on intracellular levels of glutathione (GSH), a crucial antioxidant synthesized from cysteine. A disruption of the cystine/glutamate antiporter system (System Xc^−^), which provides substrate for GSH synthesis, or a direct inhibition of GPX4 results in lipid peroxide accumulation, thereby inducing ferroptosis [3].

Mitochondria also play a significant role in ferroptosis by contributing to ROS production and metabolic regulation. Additionally, specific enzymes, such as acyl-CoA synthetase long-chain family member 4 (ACSL4), promote the incorporation of PUFAs into membrane phospholipids, further sensitizing cells to ferroptosis. The process is regulated by both transcriptional and post-transcriptional pathways. For instance, the tumor suppressor p53 and the antioxidant transcription factor Nrf2 modulate cellular responses to oxidative stress, influencing ferroptosis susceptibility [5].

Ferroptosis is classified as a type of regulated necrosis, which emphasizes its controlled nature compared to unregulated necrotic cell death caused by acute injury [3]. Moreover, ferroptosis has been found to be more immunogenic than apoptosis, meaning it can trigger a more robust immune response [6]. This immunogenicity is particularly relevant in the field of cancer treatment, where inducing ferroptosis in tumor cells may enhance the effectiveness of the anti-tumor immune response [1,3].

Research into ferroptosis has opened new therapeutic prospects, especially in oncology, where its manipulation could foster novel cancer treatments. Understanding the precise molecular mechanisms that regulate ferroptosis and its interactions with other forms of cell death remains a critical area of investigation. Numerous studies have confirmed its role in cancer biology [4,5]. Dysregulated ferroptosis has been implicated in various pathological conditions, including cancer, neurodegeneration, tissue damage, inflammation and infection [4]. The metabolic plasticity demonstrated by cancer cells provides valuable insights into the significance of metabolic reprogramming’s contribution to the tumor survival and progression [5].

CRC is one of the most prevalent malignancies of the digestive tract and ranks third in terms of mortality worldwide [7]. Interestingly, the incidence of CRC is only marginally linked to genetic or hereditary predispositions [8,9]. Despite the advancements in diagnostic methods, the treatment options for CRC remain relatively limited, frequently requiring a surgical resection of the affected colon segment, an administration of chemotherapeutic agents such as 5-FU and oxaliplatin, or a combination of these approaches. In certain cases, immunotherapy may also be considered [7,8]. For patients with disseminated CRC, adjuvant chemotherapy is typically required, which includes treatment regimens such as FOLFOX (5-FU, leucovorin and oxaliplatin), CAPOX (capecitabine and oxaliplatin), FOLFIRI (5-FU, leucovorin and irinotecan) and XELIRI (capecitabine and irinotecan). Additionally, anti-epidermal growth factor receptor (anti-EGFR) antibodies and anti-vascular endothelial growth factor receptor (anti-VEGFR) antibodies, such as bevacizumab and cetuximab, are commonly used in treatment [10]. However, a significant flaw of chemotherapy lies in its inability to target only malignant tissues, leading to collateral damage to the normal, healthy cells [7,8,9]. This lack of specificity not only diminishes treatment efficacy but also contributes to the adverse side effects, highlighting the urgent need for more effective and targeted therapeutic strategies for CRC treatment.

Recently, there has been a growing interest among researchers in the “drug repurposing” approach to discover effective anticancer therapies. This strategy involves the utilization of existing drugs, which have well-established safety profiles for treating other medical conditions [11]. In this study, we have focused on the 5-aminosalicylic acid (5-ASA), also known as mesalamine or mesalazine, which was specifically selected in accordance with the drug-repurposing concept. MES, a NSAID, inhibits lipoxygenase (LOX) and cyclooxygenase (COX) enzymes, thus reducing the production of leukotrienes and prostaglandins [12]. It has been employed for several decades in the treatment of ulcerative colitis (UC) and to maintain remission [13]. MES is available in various pharmaceutical forms, including tablets, suppositories and formulations designed for delayed or extended release, allowing the drug to act directly in the colon or be applied rectally [14]. Approximately 25% of MES is absorbed in the large intestine, while the remainder is excreted unchanged in the feces. The availability of multiple dosage forms offers the flexibility to select the most suitable formulation to achieve the desired drug concentration in the colon. MES is metabolized primarily through N-acetylation by N-acetyltransferase type 1 (NAT1) [15,16,17]. The selection of MES as a repositioning drug is supported by its relatively mild side effects compared to other NSAIDs. Furthermore, studies have demonstrated its mucosal healing properties, which are particularly relevant and beneficial for oncology patients [18,19,20]. Notably, MES exhibits antitumor activity and is commonly utilized in the chemoprevention of CRC in patients with inflammatory bowel diseases (IBDs) [13,14,16,18,21,22]. Several mechanisms underlying MES-mediated tumor growth inhibition have been explored, including the upregulation of PPAR-γ expression, EGFR inhibition, cell cycle arrest and the enhancement of replication fidelity [23,24,25,26]. Additionally, MES interacts with various components of the Wnt/β-catenin pathway, which is often disrupted in CRC [27,28]. Although the precise anticancer mechanism of MES remains incompletely understood, numerous studies have confirmed its inhibitory effect on tumor cell growth [27,28,29].

Given the promising role of ferroptosis in cancer treatment and the limited availability of its safe inducers, it is crucial to investigate whether established drugs with well-documented safety profiles can induce ferroptosis [30]. As MES is a derivative of sulfasalazine, which is recognized as a ferroptosis inducer, it is particularly important to determine whether MES shares this capability [31,32]. In this study, we evaluated the impact of MES on the expression of ferroptosis-related genes in the CRC cell line DLD-1. Furthermore, our bioinformatic analysis, using data from a microarray gene panel, revealed potential protein–protein interactions that may be involved in MES’s mechanism of action.

## 2. Materials and Methods

### 2.1. Cell Culture Conditions

Human colorectal carcinoma DLD-1 cells (ATCC^®^ CCL-221TM) were cultured in RPMI 1640 medium (cat. No. R8758; Sigma-Aldrich; Merck, St. Louis, MO, USA) supplemented with 10% fetal bovine serum (Euroclone S.p.A., Pero (MI), Italy) and 50 mg/L gentamycin (Sigma-Aldrich; Merck, St. Louis, MO, USA). Normal human colon epithelial CCD 841 CoN cell line (ATCC^®^ CRL-1790) was cultured in Minimum Essential Medium Eagle (cat. No. 12-662F; Lonza; BioWhittaker, Basel, Switzerland) supplemented with L-glutamine (ATCC, Manassas, VA, USA).

Cells were maintained at 37 °C in a 5% CO_2_ incubator (Direct Heat CO_2_; Thermo Scientific, Waltham, MA, USA). Cells were cultured in flasks (Thermo Scientific, Waltham, MA, USA) and did not exceed six passages. Once reaching 80% confluence, cells were detached using a standard trypsin–EDTA solution (Sigma-Aldrich; Merck, MO, USA) for experimental use.

### 2.2. Viability Assays

The MTT (3-[4,5-dimethylthiazol-2-yl]-2,5-diphenyltetrazolium bromide) assay (Sigma-Aldrich, St. Louis, MO, USA) was used to determine the influence of MES (item No. 70625; Cayman Chemical, Ann Arbor, MI, USA) on the DLD-1 cells’ viability. The CRC cell line was treated with different concentrations of MES (10, 20, 30, 40 and 50 mM) at pH 7.0 for 24 h. These concentrations were selected based on the available literature, where previous studies have reported similar concentrations correlating with tissue levels observed in patients receiving 2–4 g/d of 5-ASA [33].

The DLD-1 and CCD 841 CoN cells were seeded into 96-well culture plates (Thermo Scientific, Waltham, MA, USA) at a density of 10,000 cells per well. After 48 h, the cells were treated with MES solutions. MES was dissolved in the culture medium, pH was adjusted to 7.0 with NaOH and the solution was then sterile-filtered using 0.2 μm disposable syringe filters (Sartorius, Göttingen, Germany). The addition of NaOH allowed MES to dissolve at the required concentrations. MES solutions were protected from light during all stages of preparation. After 24 h of treatment, the MTT assay was performed. Briefly, MTT was dissolved in PBS to a concentration of 1 mg/mL and added to each well after the removal of MES solutions. The cells were incubated for 3 h at 37 °C in the dark, after which the MTT solution was replaced with DMSO (Sigma-Aldrich, St. Louis, MO, USA) to dissolve the formazan crystals that had formed during the incubation period.

Absorbance was measured using a BioTek Epoch Microplate Spectrophotometer (BioTek Instruments, Agilent Technologies, Santa Clara, CA, USA) at a wavelength of 570 nm, with a reference wavelength of 650 nm. The results were calculated as a fold change in the absorbance of treated cells relative to the absorbance of untreated cells.

### 2.3. Preparation of DLD-1 Cell Lysates

Cells were seeded onto 6-well plates (Thermo Scientific, Waltham, MA, USA) at a density of 400,000 cells per well. After 48 h, a 30 mM MES solution was prepared (as described above), and 3 mL of it replaced the growth medium in half of the wells. Complete growth medium served as a control. The experiment was performed in triplicate biological replicates. To determine the gene expression profile by microarray and RT-qPCR, cells were lysed after 24 h of exposure to 30 mM MES using TRIzol (Invitrogen Life Technologies, Carlsbad, CA, USA), and total RNA was isolated following the manufacturer’s instructions.

The quality of RNA extracts was assessed by electrophoresis on a 1% agarose gel, and quantitative evaluation was performed using spectrophotometric measurement with a MaestroNano MN-913 nano spectrophotometer (MaestroGen Inc., Las Vegas, NV, USA).

### 2.4. Oligonucleotide Microarray Analysis

The gene expression profile after MES treatment was analyzed using the oligonucleotide microarray method. The analysis was conducted with GeneChip™ Human Genome U133A 2.0 Array in combination with the GeneChip™ 3′ IVT PLUS Reagent Kit (Applied Biosystems, Carlsbad, CA, USA) in accordance with the manufacturer’s instructions. The arrays were scanned using the GeneChip™ Scanner 3000 System (Affymetrix, Santa Clara, CA, USA).

### 2.5. Quantitative Real-Time Polymerase Chain Reaction Assay

To validate the microarray results, real-time RT-qPCR reactions were conducted for selected genes: *SLC7A11*, *ATF3*, *HMOX1* and *CDKN1A* using the $2^{-(∆∆Ct)}$ quantification method. TATA-Box-binding protein (TBP) was used as a housekeeping gene for reference purposes in target gene expression analysis [34]. Both the target and control genes were amplified simultaneously to ensure accurate normalization and quantification of gene expression. RT-qPCR reactions were performed using the Sensi-Fast™ reagent kit (Bioline, London, UK), with the primer sequences listed as follows: *SLC7A11:* Forward, 5′ GGTTATTCCTATGTTGCGTCTC 3′; Reverse, 5′ AATAACAGCTGGTAGAGGAG 3′; *ATF3:* Forward, 5′ AGAAAGAGTCGGAGAAGC 3′; Reverse, 5′ TGAAGGTTGAGCATGTATATC 3′; *HMOX1:* Forward, 5′ CAACAAAGTGCAAGATTCTG 3′; Reverse, 5′ TGCATTCACATGGCATAAAG 3′; *CDKN1A:* Forward, 5′ CAGCATGACAGATTTCTACC 3′; Reverse, 5′ CAGGGTATGTACATGAGGAG 3′.

The real-time RT-qPCR reactions were conducted on a LightCycler^®^ 480 System (Roche, Basel, Switzerland) with a detailed thermal cycling protocol. The process began with reverse transcription at 45 °C for 10 min, followed by an initial denaturation phase at 95 °C for 2 min. This was succeeded by 45 cycles of PCR amplification, each consisting of denaturation at 95 °C for 5 s, annealing at 60 °C for 10 s, and elongation at 72 °C for 5 s. This setup ensures efficient and accurate amplification of the target gene sequences, critical for reliable quantitative analysis.

### 2.6. Bioinformatics Analysis

The data obtained from oligonucleotide microarrays were analyzed using the PL-Grid Infrastructure (available at https://www.plgrid.pl/, accessed on 16 October 2024). The analysis was conducted on the GeneSpring 13.0 platform (Agilent Technologies UK Limited, South Queensferry, UK).

Bioinformatic analysis of selected ferroptosis-related genes at the protein level was performed using the STRING online database (https://string-db.org/, accessed on 20 October 2024), with an interaction score of >0.400 as the cutoff threshold. The results of the protein–protein interactions were graphically presented.

### 2.7. Statistical Analysis

Statistical analysis was performed using STATISTICA 13.3 software (TIBCO Software Inc., Palo Alto, CA, USA), with a significance threshold set at *p* < 0.05. The qualitative data obtained from RT-qPCR were presented as the median with lower and upper quartiles, as well as minimum and maximum values. These were graphically represented using box plots.

The Shapiro–Wilk test was utilized to evaluate the normality of data distribution, while Levene’s test was applied to assess the homogeneity of variances. Given the indications from these tests for non-normal distribution and variance heterogeneity, non-parametric statistical methods were chosen. Consequently, the Kruskal–Wallis one-way ANOVA was conducted to analyze the data, followed by multiple comparison Dunn’s test as a post hoc test. These tests’ application is recommended when dealing with non-normally distributed data as they do not compare means of tested groups but their ranks. Results identified as outliers by the IQR method were omitted in the statistical analysis.

## 3. Results

### 3.1. DLD-1 Viability Assessment

**Figure 1:**
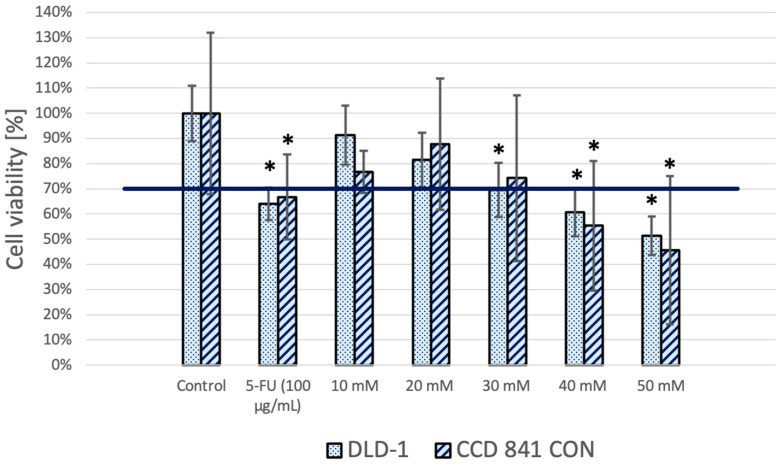
Fold change of cell viability in relation to the control group after 24 h of treatment with selected MES concentrations. 5-FU was used as a positive cytotoxicity control. Results present data obtained for DLD-1 and CCD 841 CoN cell lines, as an average of 3 biological replicates (bar) and a standard deviation of an average (whiskers). The auxiliary axis indicates the adopted level of cytotoxicity (70% viability). *—statistical significance (*p* < 0.05) versus respective control group.

A decrease in the viability of DLD-1 and CCD 841 CoN cells was observed after 24 h of treatment with MES at all tested concentrations (Figure 1). The decrease in viability was, to some extent, dose-dependent, with the lowest concentration of MES (30 mM) reducing the viability of colon cancer cells below the 70% cytotoxicity threshold. A similar reduction in the viability of normal colon cells was observed at the 30 mM MES concentration; however, this decrease was not statistically significant and did not fall below the 70% threshold. Based on these results, we selected a MES concentration of 30 mM for further analysis due to its statistically significant impact on reducing cancer cell viability, while it demonstrated only minimal effects on normal cells.

### 3.2. Differential Expression of Ferroptosis-Related Genes Based on Oligonucleotide Microarrays

During the next phase of the research, the expression of ferroptosis-related genes was compared between two groups: untreated control DLD-1 cells (DLD1_CON) and MES-treated DLD-1 cells (DLD1_MES). Gene expression profiling was carried out using an oligonucleotide microarray technique with a panel of 657 probes for ferroptosis-related genes, sourced from the GeneCards website (https://www.genecards.org; accessed 17 October 2024) and previous studies [35].

**Figure 2:**
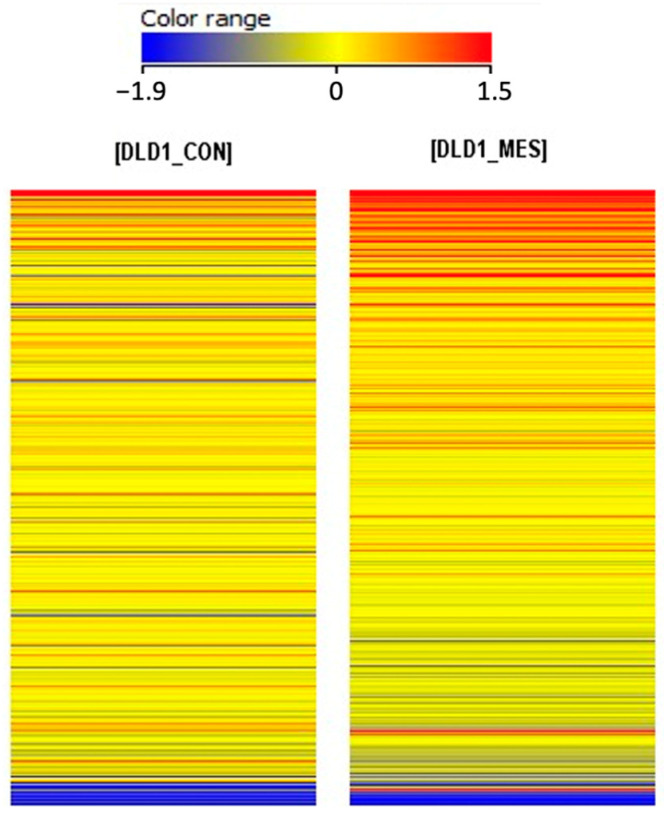
Graphical illustration of differences in expression profiles ferroptosis-related genes specific to the control DLD−1 cells (DLD1_CON) and MES−treated DLD−1 cells (DLD1_MES). The color variation between transcriptome groups indicates the presence of differences in gene expression profiles. Red—higher signal, high gene expression; blue—lower signal, low gene expression.

The differentiation of 657 probes for ferroptosis-related genes between the studied groups was evaluated using the heatmap analysis in GeneSpring 13.0 XG software (Figure 2). Changes in gene expression were assessed based on the color shifts of the fluorescence signals, with an increase in expression indicated by a transition towards red and a decrease in expression represented by a shift towards blue.

A *t*-test was performed to identify genes related to ferroptosis that were differentially expressed between the control and MES-treated groups. The results were visualized in a volcano plot to emphasize the genes that showed significant changes. The comparison of transcriptomic profiles between MES-treated DLD-1 cells and control cells revealed 84 probes with statistically significant expression differences, corresponding to 56 genes (Table 1).

To further characterize ferroptosis-related genes in each study group, the fold change (FC) parameter was calculated, representing the log2 difference in fluorescence signals between groups and indicating the direction of the observed changes (Table 2).

Among the 84 probes that exhibited a statistically significant change greater than two-fold, 44 genes were upregulated, and 12 genes were downregulated in cells treated with MES.

**Figure 3:**
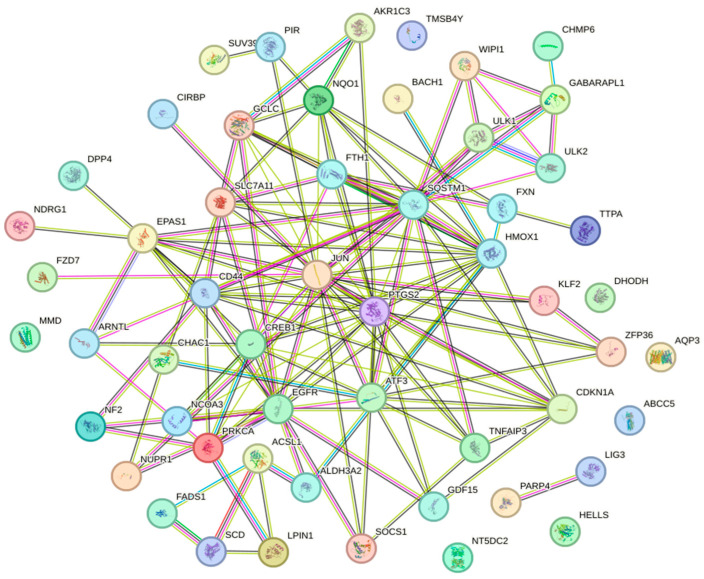
Network of protein–protein interactions generated using the STRING database (Search Tool for the Retrieval of Interacting Genes/Proteins).

Next, the relationships between the differentially expressed genes were analyzed using a bioinformatic approach with the STRING database. The resulting network of potential protein–protein interactions consisted of 139 edges and 54 nodes (*p* < 0.001, mean confidence = 0.400) (Figure 3). In this network, edges represent protein interactions, and the edge weight indicates the likelihood of these interactions occurring.

Subsequent analysis with the Kyoto Encyclopedia of Genes and Genomes (KEGG) and STRING databases revealed that the selected genes play significant roles in biological processes and are involved in 86 signaling pathways, many of which are associated with tumorigenic processes (Table 3).

**Figure 4:**
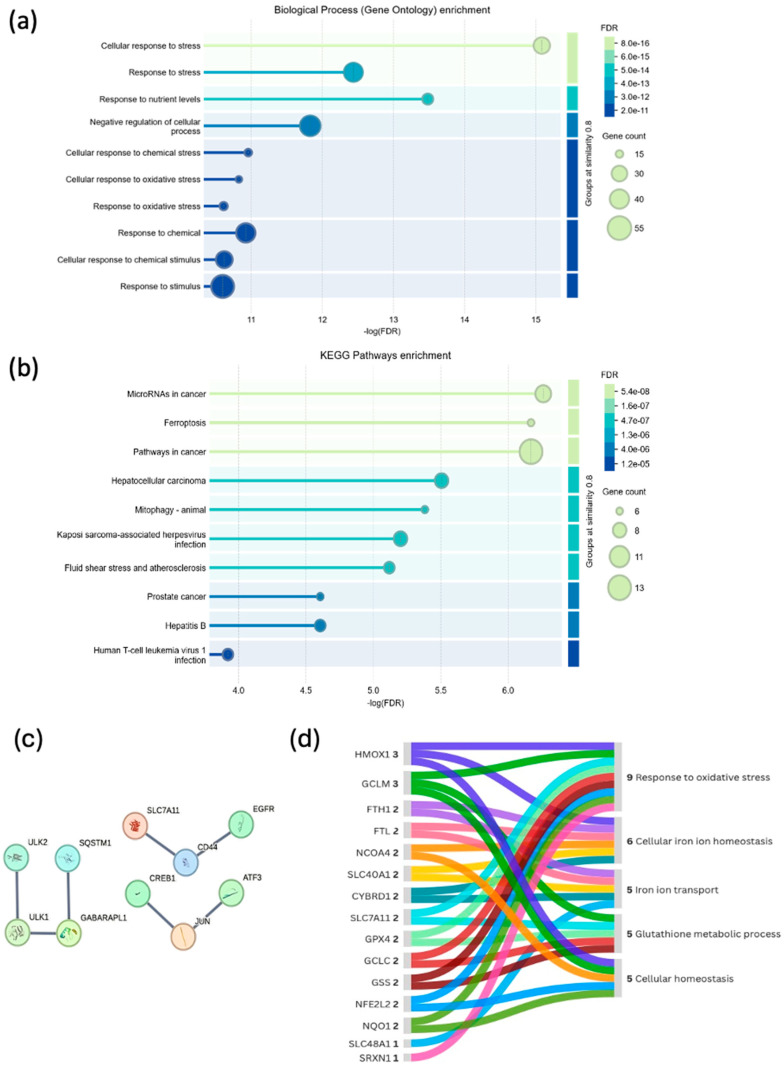
Enriched analysis of genes associated with ferroptosis includes the following: (**a**) a bubble plot of biological processes; (**b**) a bubble plot of molecular pathways; (**c**) a STRING network presenting interactions with the highest confidence level of 0.99; (**d**) an illustration of gene involvement in specific processes associated with ferroptosis.

An enrichment analysis of the ferroptosis-related genes regulated by MES was conducted using the STRING database. Ten biological processes were identified, with the majority of genes involved in the cellular stress response and nutrient level response (Figure 4a). Additionally, ten cancer-related signaling pathways were highlighted, with the largest number of genes involved in miRNA regulation in cancer and ferroptosis (Figure 4b). We also determined which genes interact with each other, setting the highest confidence index at 0.99 (Figure 4c). Furthermore, an in-depth analysis of genes involved in specific processes associated with ferroptosis was conducted (Figure 4d).

**Figure 5:**
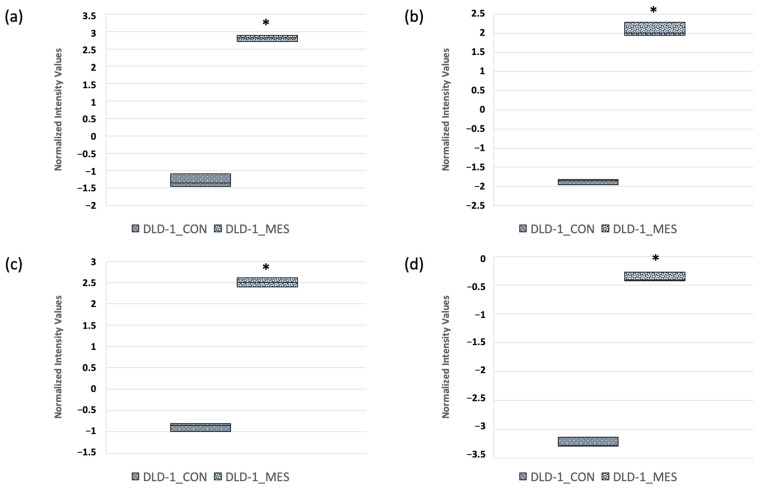
Expression profiles of *SLC7A11* (**a**), *ATF3* (**b**), *HMOX1* (**c**) and *CDKN1A* (**d**) genes in DLD−1 cells after 24 h of 30 mM MES treatment obtained by oligonucleotide microarray. Results are presented as the median with the 25–75% range, and minimum and maximum values. *—statistical significance (*p* < 0.05) versus control; [DLD1_CON]—control DLD−1 cells, [DLD1_MES]—MES-treated DLD−1 cells.

Based on the results of the oligonucleotide microarray analysis, we identified 84 differentially expressed ferroptosis-related transcripts. However, we chose to further validate only those with the highest FC values—*SLC7A11*, *ATF3*, *HMOX1* and *CDKN1A*—using an independent real-time RT-qPCR method (Figure 5). Additionally, these genes are known to play key roles in the ferroptosis process [36,37,38].

### 3.3. MES Alternates the Expression Level of SLC7A11, ATF3, HMOX1 and CDKN1A

**Figure 6:**
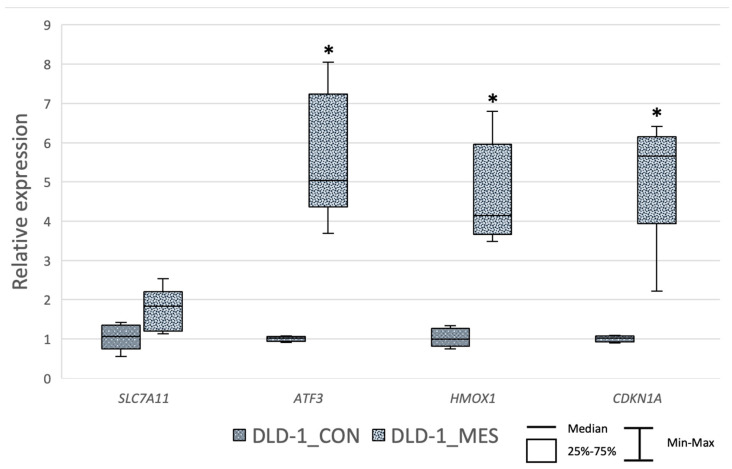
Relative gene expression of selected genes in DLD−1 cells after 24 h of 30 mM MES treatment. Results are presented as the median (horizontal line) with the 25–75% range (box) and minimum and maximum values (whiskers). *—statistical significance (*p* < 0.05) versus control; [DLD1_CON]—control DLD−1 cells, [DLD1_MES]—MES-treated DLD−1 cells. Sample size—three biological and three technical replicates for each test group.

Treatment with 30 mM MES for 24 h resulted in the upregulation of all selected genes (Figure 6). The median fold changes were 1.82 for *SLC7A11*, 5.03 for *ATF3*, 4.13 for *HMOX1* and 5.65 for *CDKN1A*. However, a statistically significant increase in expression was observed for the *ATF3* (*p* = 0.005), *HMOX1* (*p* = 0.030),and *CDKN1A* (*p* = 0.011) genes when comparing MES-treated cells to the control group.

## 4. Discussion

CRC, one of the most commonly diagnosed cancers, continues to pose a serious threat despite advances in medical science and an expansion of treatment options [39]. Originating from the intestinal epithelial cells, CRC develops through sequential genetic and epigenetic mutations that lead to hyperproliferation, adenoma, carcinoma and ultimately metastasis [40]. Several well-known abnormalities in signaling pathways contribute to the development and progression of this cancer, with the most common being the Wnt/β-catenin, P53, transforming growth factor-beta (TGF-beta) and epidermal growth factor receptor (EGFR) pathways [41].

A significant challenge in treating CRC lies in its high recurrence rate; approximately 20% of patients experience a recurrence post-surgery, necessitating adjuvant therapy to reduce the risk of relapse [42].

Given the urgent need for effective therapies in CRC, various approaches are being explored, including drug repositioning. Drug repositioning involves testing existing drugs for alternative therapeutic applications. Compared to developing new drugs from scratch, the primary advantage of this approach is the significantly reduced time and risk involved in bringing the drug to market. Drugs subjected to repositioning are already well characterized regarding their pharmacokinetics, pharmacodynamics and side effects. Once a new potential use is identified for an existing drug, it can quickly progress to Phase I or Phase II pre-clinical studies [43]. Notably, several repositioned drugs, such as aspirin [44], metformin [45] and sirolimus [46], show promise in treating CRC.

MES, utilized in our study, is a repositioned drug that has demonstrated inhibitory effects on cancer cells in previous studies [25,27,33]. However, its impact on the ferroptosis process remains underexplored, with limited data available.

In 2012, Dixon et al. [47] discovered that the pharmacological inhibition of cystine uptake mediated by solute carrier family 7 member 11 (SLC7A11), using compounds like erastin, induces this novel form of cell death. It is worth noting that cysteine, derived from cystine, is crucial for GSH synthesis—a key antioxidant in cells. Consequently, the pharmacological inhibition of SLC7A11 induces oxidative stress and lipid peroxidation, leading to cell death [36]. Despite this, well-known SLC7A11 inhibitors such as sulfasalazine and erastin are rarely used in clinical settings due to their off-target effects [48].

Ferroptosis plays a dual role depending on the disease context, potentially exacerbating or inhibiting the condition. However, in CRC, inducing ferroptosis has been shown to inhibit both tumor development and metastasis [30]. This recently characterized form of cell death has garnered significant attention within the scientific community for its potential to halt the proliferation of colon cancer cells [49]. As the process of ferroptosis offers hope for new implications in CRC therapy, new ferroptosis inducers are still under investigation [50]. Nevertheless, a drug with a well-established therapeutic profile has a significant advantage due to its known pharmacokinetics and pharmacodynamics. MES, as a repositioned drug, has the potential to be promptly directed into clinical trials, where, as a ferroptosis inducer, a novel CRC treatment approach may be supported. In 2025, four studies investigating MES and colorectal diseases were reported, three of which specifically focused on CRC. Relevant information was retrieved from the ClinicalTrials.gov database (accessed 10 January 2025) and is summarized in Table 4.

Preliminary results from clinical trials, including MesaCAPP and related studies, provide promising evidence for mesalazine’s potential role in the chemoprevention and treatment of CRC. These trials evaluated mesalazine’s effects on gene expression, inflammation and tumor progression, further supporting its potential utility in clinical applications.

There are only a few reports on the association of MES with ferroptosis. Therefore, our study aimed to evaluate the expression profile of ferroptosis-related genes following treatment with MES in an in vitro model.

We initially observed that MES reduced the viability of DLD-1 tumor cells in a dose-dependent manner, showing significant cytotoxicity at a concentration of 30 mM, while exerting minimal effects on normal intestinal cells. Moreover, the concentrations used in the study, ranging from 10 to 40 mM, correspond to the tissue concentrations observed in patients receiving daily doses of 2, 3 or 4 g of MES. Given this context, we chose a 30 mM concentration for further analysis to evaluate its effects in a manner that reflects likely clinical conditions [33]. These findings align with prior studies demonstrating MES’s tumor growth-inhibiting properties [29,51]. To delve deeper, we conducted a microarray analysis, and we identified 56 ferroptosis-associated genes with a fold change (FC) greater than 2.0. The expression profiles of these genes were subsequently analyzed using the STRING database to predict potential protein–protein interactions.

**Figure 7:**
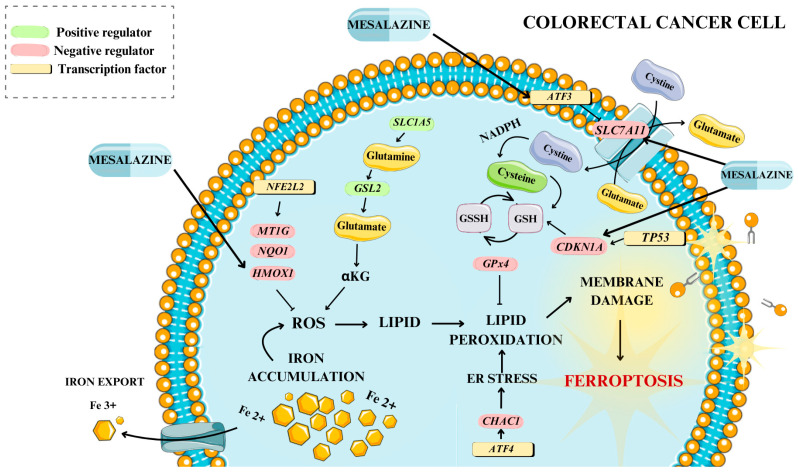
A description of key genes, including *ATF3*, *SLC7A11*, *HMOX1* and *CDKN1A*, along with MES—modulated signaling pathways involved in ferroptosis in CRC. The figure was partly generated using Servier Medical Art, provided by Servier and licensed under a Creative Commons Attribution 3.0 unported license.

From this group, we selected four genes—*SLC7A11*, *ATF3*, *HMOX1* and *CDKN1A*—for detailed analysis as they showed the highest fold changes, each exceeding 7. These genes were subsequently validated at the mRNA level through real-time RT-qPCR in two sample groups: cells treated with 30 mM MES and untreated control cells. We focused on these genes due to their critical roles in ferroptosis: *SLC7A11* is essential for regulating cystine uptake and GSH biosynthesis, both pivotal in ferroptosis development [36]. *ATF3* and *HMOX1* are key players in the cellular response to oxidative stress, a major element of ferroptosis [37,38]. *CDKN1A* (p21) is involved in cell cycle regulation and modulates cellular antioxidant responses, vital in the context of ferroptosis [52]. These genes have also been shown in previous research to play significant roles in cancer biology and therapy resistance, making them highly relevant targets for a study involving a potential therapeutic agent like MES [53,54,55,56,57]. Figure 7 illustrates the proposed key genes and pathways influenced by MES, as well as their roles in the process of ferroptosis in CRC cells.

### 4.1. Markers of Ferroptosis PTGS2 and CHAC1 Overexpressed After MES Treatment

*PTGS2* encodes prostaglandin-endoperoxide synthase 2, an enzyme primarily responsible for converting arachidonic acid into prostaglandins. One of its products, Prostaglandin E2, influences several cellular signaling pathways involved in proliferation, apoptosis and angiogenesis [58]. Moreover, PTGS2 has been linked to the process of ferroptosis. Research by Yang et al. [6] demonstrated that *PTGS2* is the most upregulated gene among 83 oxidative-stress-related genes in BJeLR cells treated with erastin or RSL3. Consequently, *PTGS2* has been identified as a biomarker of ferroptosis, both in vivo and in vitro [59].

CHAC1, or glutathione-specific gamma-glutamylcyclotransferase 1, plays a crucial role in reducing intracellular GSH levels by converting GSH into the dipeptide 5-oxoproline and cysteinylglycine [60]. The degradation of GSH mediated by CHAC1 is an important trigger of ferroptosis induced by, among others, erastin. Therefore, a significant regulator of oxidative stress, CHAC1 is also recognized as a marker of ferroptosis. It influences calcium signaling and mitochondrial function by degrading GSH. Furthermore, *CHAC1* expression is regulated by the transcription factors ATF3 and ATF4 [60,61].

In our research, we have observed an increase in the expression of ferroptosis markers, specifically *PTGS2* and *CHAC1*, suggesting that MES may induce ferroptosis. The literature on MES-induced ferroptosis is sparse; however, a recent study by Ye and Liu [62] supports our hypothesis that MES can trigger this process. While their findings suggested that MES-induced ferroptosis may negatively affect the healing process in ulcerative colitis patients, these results highlight the potential of MES to induce ferroptosis [62].

It should be noted that ferroptosis is gaining increasing attention in CRC research due to its ability to reduce cancer cells resistance to chemotherapy and directly induce cancer cell death [30].

### 4.2. SLC7A11, ATF3/CHAC1 and GSH Levels in Colon Cancer Cell

SLC7A11 plays a crucial role in the ferroptosis process through the Xc^−^ system [48]. Cysteine, an essential amino acid for cancer cells, is required to maintain cellular redox balance and for GSH synthesis. The primary source of cysteine for these cells is extracellular cystine, which is transported into the cell by SLC7A11. Once inside the cell, cystine is reduced to two cysteine molecules through a reaction involving NADPH. GSH, a tripeptide composed of cysteine, glutamate and glycine, plays a vital role in maintaining cellular redox balance, and its levels are limited by the availability of cysteine. In its reduced form, GSH serves as a cofactor for ROS-detoxifying enzymes, protecting cells from oxidative damage. The inhibition of SLC7A11 by erastin reduces cystine uptake into the cell and impairs GSH synthesis, leading to a disruption in redox balance, lipid peroxidation and ultimately ferroptotic cell death [36,48].

Although our study’s microarray analysis showed an expression change in the *SLC7A11* gene, this finding was not confirmed by RT-qPCR. However, other genes showed a strong correlation with GSH levels, which is closely associated with the process of ferroptosis [36].

Activating Transcription Factor 3 (ATF3) is a member of the ATF/cAMP response element-binding (CREB) family of transcription factors. ATF3 plays a critical role in various cellular processes, including immunogenicity, metabolism and oncogenesis [63].

As reported by Bottone et al. [64], *ATF3* expression was found to be downregulated in colon tumors compared to the surrounding normal tissue. The researchers demonstrated that *ATF3* expression increases following treatment with NSAIDs, troglitazone, diallyl disulfide and resveratrol. The overexpression of *ATF3* was shown to inhibit migration and the invasion of HCT116 cells, as well as reduce the size of mouse tumor xenografts [64].

In a study by Wang et al. [65], ferroptosis was activated by artesunate in Burkitt’s lymphoma (BL) cells, resulting in the activation of the *ATF4-CHOP-CHAC1* pathway, with *CHAC1* being overexpressed. As previously mentioned, the upregulation of *CHAC1* contributes to a significant reduction in GSH levels, which increases the cell’s susceptibility to lipid peroxidation and oxidative stress. Furthermore, silencing *CHAC1* expression in BL cells leads to increased resistance to ferroptosis and enhanced cell viability, suggesting that *CHAC1* overexpression acts as a trigger for cell death in this context [60].

In our study, both *CHAC1* and *ATF3* were overexpressed, which could serve as important triggers for MES-induced ferroptosis. ATF3 is known to regulate the transcription of *CHAC1,* and the upregulation of *CHAC1* leads to a significant decrease in GSH levels. This reduction in GSH can contribute to the disruption of the cell’s redox balance, making the cell more susceptible to ferroptotic cell death.

### 4.3. HMOX1 as a Contributor to Excess Iron Ions in the Cell

Heme oxygenase 1 (HMOX1) is an enzyme that plays a key role in cellular metabolism by catalyzing the degradation of heme into biliverdin-IXα (BV), carbon monoxide (CO) and ferrous iron (Fe^2+^) [38]. *HMOX1* has been associated with CRC, where its expression levels can have varying effects depending on the stage of the disease [38].

Andres et al. [53] demonstrated that CRC patients with HMOX1-positive tumor tissues had significantly higher survival rates compared to those whose tumors were HMOX1-negative. A high expression of *HMOX1* can lead to the Fenton reaction due to increased levels of ferrous iron, which may contribute to oxidative stress, especially in cells with insufficient free radical scavengers [66].

In our study, MES treatment led to the overexpression of *HMOX1*. Given its key role in iron metabolism, this overexpression may lead to the accumulation of Fe^2+^ in cancer cells, potentially triggering the induction of ferroptosis.

### 4.4. CDKN1A Overexpression After MES Treatment

*CDKN1A* encodes the cyclin-dependent kinase inhibitor p21, which is crucial for the negative regulation of cell cycle progression and gene expression [67]. A loss or downregulation of p21 can lead to abnormal cell proliferation and tumorigenesis. Additionally, studies have shown that reduced *CDKN1A* expression is associated with increased metastatic potential and lower patient survival rates [67].

Tarangelo et al. [68] demonstrated that in cancer cells, wild-type p53-regulated *CDKN1A* can delay the onset of ferroptosis under cysteine-deficient conditions. Furthermore, p53-mediated activation of CDKN1A/p21-regulated GSH metabolism can inhibit cellular ferroptosis [68]. Our findings are partially consistent with this research; MES treatment resulted in an increase in *CDKN1A* expression, suggesting a potential delay or inhibition of ferroptosis. However, since the DLD-1 line carries a mutant type *p53*, the response pathway in our study may differ [69].

### 4.5. Wnt/β-Catenin Pathway Inhibition Can Result in Induction of Ferroptosis

The Wnt/β-catenin pathway, often disrupted in CRC patients, plays a crucial role in tumor invasion, progression and metastasis. Targeting this pathway is, therefore, a promising strategy for the treatment of CRC [70].

MES has been studied extensively for its ability to inhibit the Wnt/β-catenin pathway through multiple mechanisms, including the inhibition of protein phosphatase 2A, interference with TCF4/β-catenin interactions, and reduction in nuclear β-catenin levels. These studies collectively demonstrate that MES can effectively reduce the expression of Wnt target genes, highlighting its potential therapeutic value in CRC management [21,27,28].

Fascinatingly, recent studies also suggest an interplay between the inhibition of the Wnt/β-catenin pathway and the induction of ferroptosis [71]. Wang et al. [72] demonstrated that the activation of Wnt/β-catenin signaling in gastric cancer cells suppressed ferroptosis. This occurs through the direct interaction of the β-catenin/TCF4 transcriptional complex with the GPX4 promoter, which enhances *GPX4* expression and consequently inhibits ferroptosis [72]. In this context, our findings are consistent with previous studies that indicate an inhibitory effect of MES on the Wnt/β-catenin pathway. Furthermore, our results suggest that MES may play a significant role as a compound capable of inducing ferroptosis.

### 4.6. Comparison of Mesalazine with Known Inducers of Ferroptosis

Several compounds have been previously investigated for their potential to induce ferroptosis in CRC cells [73,74,75]. These include novel substances [74,76], drugs currently used in CRC treatment [75], and repositioned drugs as described earlier [46,73]. A well-known inducer of ferroptosis is erastin, which primarily mediates ferroptosis through the Xc^−^ system. However, its clinical application is hindered by limited water solubility and unstable metabolism in the human body [77].

New natural-origin compounds, such as tagitinin C and talaroconvolutin, have also been investigated for their ferroptosis-inducing properties in CRC cells [74,76]. Xia et al. [76] demonstrated that talaroconvolutin, a compound isolated from the endophytic fungus *Talaromyces purpureogenus*, is even more effective in inducing ferroptosis than erastin.

It has also been shown that the combination of dihydroartemisinin (DHA) and pyropheophorbide iron induces ferroptosis and increases the sensitivity of colorectal cancer cells to anti-PD-L1 immunotherapy [78].

In turn, elesclomol, by promoting copper-dependent ferroptosis, effectively targets colorectal cancer cells through the degradation of the copper transporter ATP7A [79]. Additionally, cancer cell proliferation was suppressed by iron oxide hydroxide nanospheres and zinc oxide nanospheres, which triggered ferroptosis [80].

Moreover, cetuximab was found to enhance RSL3-induced ferroptosis in colorectal cancer cells by inhibiting the p38/NRF2/HMOX1 signaling pathway [75]. The efficacy of cetuximab was further increased by β-elemene and vitamin C, both of which induced ferroptosis, enhancing cancer cell sensitivity to treatment [81].

Representatives of NSAIDs such as aspirin and sulfasalazine have also been studied in this context. Both have demonstrated the ability to induce ferroptosis, with sulfasalazine emerging as a key ferroptosis inducer [73,82]. Interestingly, MES, a derivative of sulfasalazine, has gained broader therapeutic use compared to its parent compound due to its milder side effects [31].

In our study, MES, an established drug, was highlighted in a new light as an inducer of ferroptosis in colorectal cancer cells. Compared to the compounds mentioned above, MES offers several advantages, further supporting its potential as a therapeutic agent. First, compared to newly isolated compounds such as tagitinin C and thalaroconvolutin, MES is an FDA-approved drug with well-established pharmacokinetics and pharmacodynamics. This familiarity facilitates its direct inclusion in clinical trials and further testing. Second, compared to drugs in the cytostatic group, MES has significantly milder side effects. Unlike conventional chemotherapy, which often causes adverse effects on healthy tissues, MES not only spares normal tissues but also demonstrates regenerative properties [19,20].

These combined advantages position MES as a promising new ferroptosis inducer, offering clear benefits over currently known inducers.

In conclusion, our study highlights MES, a drug well-established for its safety and noted anticancer properties [16], as a potent inducer of ferroptosis. MES exposure in colon cancer cells resulted in changes in the expression of 56 ferroptosis-related genes, including ferroptosis markers such as *PTGS2* and *CHAC1*. These findings have significant clinical implications as the induction of ferroptosis could play a crucial role in overcoming chemotherapeutic drug resistance, targeted therapy resistance and immunotherapy resistance [30]. It is also worth noting that colorectal cancer stem cells (CCSc), largely responsible for CRC recurrence, are more sensitive to ferroptosis than normal cells [83]. The specificity of MES in targeting cancer cells could lead to comprehensive tumor eradication and help to prevent recurrence. Additionally, its capacity to promote mucosal healing and reduce tumor cell viability positions MES as an advantageous candidate for combination chemotherapy. This could enhance the regeneration of healthy tissues and increase the overall efficacy of cancer treatments.

### 4.7. Limitations

Our research was conducted in an in vitro model, and, while it provides important insights into the molecular mechanisms, these findings need to be validated in vivo to confirm their clinical relevance. Although we focused on gene expression analysis, further confirmation of our findings at the protein level would provide a more comprehensive understanding of MES’s mechanisms and its impact on ferroptosis. Moreover, we primarily focused on the short-term effects of MES treatment. Long-term studies could offer valuable insights into its sustained impact on colon cancer cells, including potential resistance mechanisms. The MES concentration used in this study (30 mM) was based on previously reported tissue concentrations. Nonetheless, further research is necessary to determine the precise therapeutic window and dosing that could translate to clinical settings. Additionally, investigating specific signaling pathways, such as the PI3K/Akt pathway and the NRF2-mediated oxidative stress response, which are influenced by MES, will provide a deeper understanding of its mechanisms.

## 5. Conclusions

Our study demonstrated that MES significantly reduces the viability of CRC cells in the DLD-1 line, with ferroptosis likely serving as the mechanism of cell death following treatment. Investigating the influence of MES on ferroptosis-related genes, especially *ATF3*, *HMOX1* and *CDKN1A*, may provide valuable insights into the molecular mechanisms underlying its action and offer potential therapeutic strategies for cancer treatment. Notably, MES is a well-established compound with a robust safety profile, having been used in the pharmaceutical market for many years. Given the growing interest in ferroptosis inducers as a novel strategy for CRC treatment, our findings could offer valuable insights for other healthcare professionals and researchers in the field. However, it is important to acknowledge that the results observed in vitro may not directly translate to in vivo outcomes, highlighting the need for further research to confirm these effects in clinical settings.

Additionally, future studies should include a direct comparison of MES with other established ferroptosis inducers to better contextualize its effects. Moreover, our findings should be validated at the protein level to provide a more comprehensive understanding of MES’s impact on ferroptosis.

## Figures and Tables

**Table 1:** The number of probes differentiating the studied groups based on the statistical assumptions applied.

** 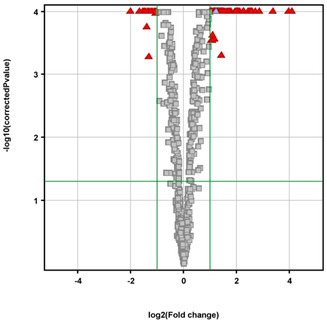 **		***p*** **All**	***p*** **< 0.05**	***p*** **< 0.02**	***p*** **< 0.01**	***p*** **< 0.005**	***p*** **< 0.001**
	DLD1_MES vs. DLD1_CON						
	FC all	657	359	318	292	261	203
	FC > 1.1	452	359	318	292	261	203
	FC > 1.5	169	169	167	166	165	153
	FC > 2.0	84	84 *	84	84	84	84
	FC > 3.0	26	26	26	26	26	26

FC—fold change, *—statistical significance (*p* < 0.05), and FC > 2.0 are represented by green axes, [DLD1_CON]—control DLD−1 cells, [DLD1_MES]—MES-treated DLD−1 cells. The volcano plot—red triangles are significant differentially expressed genes; grey squares are nonsignificant genes.

**Table 2:** Characteristics of ferroptosis-related genes, which had more than a two-fold statistically significant change in their expression.

Probe Number	Gene Symbol	FC (abs)	Alteration of Expression DLD1_MES vs. DLD1_CON
**217678_at**	***SLC7A11***	**16.91**	*↑*
**202672_s_at**	***ATF3***	**15.52**	*↑*
**203665_at**	***HMOX1***	**10.44**	*↑*
**202284_s_at**	***CDKN1A***	**7.30**	*↑*
201465_s_at	*JUN*	6.60	*↑*
200632_s_at	*NDRG1*	5.99	*↑*
221577_x_at	*GDF15*	5.89	*↑*
210001_s_at	*SOCS1*	5.65	*↑*
209160_at	*AKR1C3*	5.54	*↑*
209921_at	*SLC7A11*	5.39	*↑*
201464_x_at	*JUN*	4.86	*↑*
204748_at	*PTGS2*	4.77	*↑*
201466_s_at	*JUN*	4.33	*↑*
210544_s_at	*ALDH3A2*	4.17	*↑*
207469_s_at	*PIR*	4.14	*↑*
213281_at	*JUN*	4.03	*↑*
217150_s_at	*NF2*	3.97	*↓*
39248_at	*AQP3*	3.95	*↑*
207528_s_at	*SLC7A11*	3.85	*↑*
200878_at	*EPAS1*	3.44	*↑*
202054_s_at	*ALDH3A2*	3.34	*↑*
219371_s_at	*KLF2*	3.22	*↑*
213632_at	*DHODH*	3.19	*↓*
208869_s_at	*GABARAPL1*	3.12	*↑*
204194_at	*BACH1*	3.12	*↑*
211458_s_at	*GABARAPL3*	3.11	*↑*
213112_s_at	*SQSTM1*	2.97	*↑*
219270_at	*CHAC1*	2.96	*↑*
204123_at	*LIG3*	2.92	*↓*
211017_s_at	*NF2*	2.90	*↓*
202923_s_at	*GCLC*	2.87	*↑*
209380_s_at	*ABCC5*	2.81	*↑*
213093_at	*PRKCA*	2.81	*↑*
204991_s_at	*NF2*	2.78	*↓*
214211_at	*FTH1*	2.76	*↑*
212276_at	*LPIN1*	2.73	*↑*
201468_s_at	*NQO1*	2.72	*↑*
218743_at	*CHMP6*	2.71	*↓*
209230_s_at	*NUPR1*	2.71	*↑*
212274_at	*LPIN1*	2.65	*↑*
201467_s_at	*NQO1*	2.65	*↑*
207348_s_at	*LIG3*	2.62	*↓*
208868_s_at	*GABARAPL1*	2.60	*↑*
202239_at	*PARP4*	2.57	*↑*
211162_x_at	*SCD*	2.56	*↓*
201471_s_at	*SQSTM1*	2.53	*↑*
207275_s_at	*ACSL1*	2.49	*↑*
211708_s_at	*SCD*	2.49	*↓*
202922_at	*GCLC*	2.47	*↑*
211092_s_at	*NF2*	2.46	*↓*
212063_at	*CD44*	2.41	*↑*
203827_at	*WIPI1*	2.39	*↑*
203706_s_at	*FZD7*	2.37	*↑*
203705_s_at	*FZD7*	2.37	*↑*
210614_at	*TTPA*	2.36	*↓*
221576_at	*GDF15*	2.34	*↑*
201531_at	*ZFP36*	2.34	*↑*
220085_at	*HELLS*	2.34	*↓*
209824_s_at	*ARNTL*	2.29	*↑*
213836_s_at	*WIPI1*	2.28	*↑*
211091_s_at	*NF2*	2.28	*↓*
204062_s_at	*ULK2*	2.27	*↑*
208962_s_at	*FADS1*	2.25	*↑*
218051_s_at	*NT5DC2*	2.25	*↓*
209333_at	*ULK1*	2.2	*↑*
204312_x_at	*CREB1*	2.17	*↑*
207700_s_at	*NCOA3*	2.17	*↑*
210818_s_at	*BACH1///GRIK1-AS2*	2.15	*↑*
211478_s_at	*DPP4*	2.14	*↑*
218619_s_at	*SUV39H1*	2.13	*↓*
209061_at	*NCOA3*	2.11	*↑*
201963_at	*ACSL1*	2.10	*↑*
210519_s_at	*NQO1*	2.09	*↑*
215195_at	*PRKCA*	2.09	*↑*
211352_s_at	*NCOA3*	2.08	*↑*
205565_s_at	*FXN*	2.08	*↓*
206769_at	*TMSB4Y*	2.07	*↓*
200811_at	*CIRBP*	2.05	*↓*
202053_s_at	*ALDH3A2*	2.05	*↑*
203414_at	*MMD*	2.05	*↑*
201983_s_at	*EGFR*	2.04	*↑*
202644_s_at	*TNFAIP3*	2.03	*↑*
210971_s_at	*ARNTL*	2.02	*↑*
214513_s_at	*CREB1*	2.01	*↑*

FC—fold change, abs—absolute value; ↑—gene overexpression, ↓—gene down expression, [DLD1_CON]—control DLD−1 cells, [DLD1_MES]—MES-treated DLD−1 cells. *p* value for each probe was <0.001. Sample size—three biological replicates for each test group, bold—differential transcripts whose expression was validated by RT-qPCR.

**Table 3:** Selected biological processes and signaling pathways in which specific differentially expressed genes play a significant role.

Biological Process	Genes
Cellular response to stress	*HMOX1*, *KLF2*, *WIPI1*, *EPAS1*, *EGFR*, *SLC7A11*
Regulation of metabolic process	*HMOX1*, *KLF2*, *GDF15*, *MMD*, *WIPI1*, *EPAS1*
Regulation of developmental process	*HMOX1*, *KLF2*, *GDF15*, *TTPA*, *MMD*
Regulation of cell death	*HMOX1*, *EGFR*, *SLC7A11*, *CHMP6*, *NQO1*
Regeneration	*HMOX1*, *FZD7*, *ULK1*, *JUN*, *CDKN1A*
Regulation of epithelial cell proliferation	*HMOX1*, *EGFR*, *FZD7*, *JUN*, *NUPR1*
Selective autophagy	*ULK1*, *SQSTM1*, *ULK2*, *GABARAPL1*
Fatty acid metabolic process	*FADS1*, *ALDH3A2*, *PTGS2*, *SCD*, *AKR1C3*
Iron ion homeostasis	*HMOX1*, *EPAS1*, *FTH1*, *FXN*
Glutathione transmembrane transport	*SLC7A11*, *ABCC5*
Cyclooxygenase pathway	*PTGS2*, *AKR1C3*
**Pathway**	**Genes**
Ferroptosis	*HMOX1*, *TP53*, *FTH1*, *SLC7A11*, *ACSL1*, *GCLC*
mTOR signaling pathway	*FZD7*, *ULK1*, *ULK2*, *LPIN1*, *PRKCA*
Oxytocin signaling pathway	*EGFR*, *PTGS2*, *JUN*, *CDKN1A*, *PRKCA*
HIF-1 signaling pathway	*HMOX1*, *EGFR*, *CDKN1A*, *PRKCA*
TNF signaling pathway	*PTGS2*, *JUN*, *CREB1*, *TNFAIP3*
FoxO signaling pathway	*KLF2*, *EGFR*, *CDKN1A*, *GABARAPL1*
AMPK signaling pathway	*ULK1*, *SCD*, *CREB1*
Wnt signaling pathway	*TP53*, *FZD7*, *JUN*, *PRKCA*
PI3K-Akt signaling pathway	*TP53*, *EGFR*, *ATF4*, *CDKN1A*, *CREB1*, *PRKCA*
JAK-STAT signaling pathway	*EGFR*, *CDKN1A*, *SOCS1*
MAPK signaling pathway	*TP53*, *EGFR*, *ATF4*, *JUN*, *PRKCA*
IL-17 signaling pathway	*PTGS2*, *JUN*, *TNFAIP3*

**Table 4:** Selected clinical trials of MES-based gene therapy at CRC at https://clinicaltrials.gov (accessed 10 January 2025).

Research Title	Application Route	Status	NCT Number	Participants
Chemopreventive Action of Mesalazine on Colorectal Cancer: A Pilot Study for an “in Vivo” Evaluation of the Molecular Effects on β-catenin Signaling Pathway	Mesalazine 800 mg orally, three times daily for 3 months.	Completed	NCT02077777	21
Mesalamine for Colorectal Cancer Prevention Program in Lynch Syndrome (MesaCAPP)	Mesalazine 2400 mg or 1200 mg mesalamine total, once daily in the morning for the treatment phase of the study (24 months).	Terminated (due to poor patient recruitment and insufficient funding)	NCT03070574	8
Mesalamine for Colorectal Cancer Prevention Program in Lynch Syndrome (MesaCAPP)	Mesalazine 2 g once daily for 2 years.	Recruiting	NCT04920149	150

## Data Availability

The data that support the findings of this study are available from the corresponding author upon reasonable request.
